# Long‐term white matter tract reorganization following prolonged febrile seizures

**DOI:** 10.1111/epi.13724

**Published:** 2017-03-23

**Authors:** Suresh S. Pujar, Kiran K. Seunarine, Marina M. Martinos, Brian G. R. Neville, Rod C. Scott, Richard F. M. Chin, Chris A. Clark

**Affiliations:** ^1^ Neurosciences UnitUCL Great Ormond Street Institute of Child HealthLondonUnited Kingdom; ^2^ Great Ormond Street Hospital for Children NHS Foundation TrustLondonUnited Kingdom; ^3^ Young EpilepsyLingfieldSurreyUnited Kingdom; ^4^ Imaging and Biophysics UnitUCL Great Ormond Street Institute of Child HealthLondonUnited Kingdom; ^5^ Developmental Cognitive Neuroscience UnitUCL Great Ormond Street Institute of Child HealthLondonUnited Kingdom; ^6^ Department of Neurological SciencesUniversity of Vermont College of MedicineBurlingtonVermontU.S.A; ^7^ Muir Maxwell Epilepsy CentreDepartment of Child Life and HealthThe University of EdinburghEdinburghUnited Kingdom

**Keywords:** Status epilepticus, White matter, Axonal injury, Neuroplasticity, Diffusion tensor imaging

## Abstract

**Objective:**

Diffusion magnetic resonance imaging (MRI) studies have demonstrated acute white matter changes following prolonged febrile seizures (PFS), but their longer‐term evolution is unknown. We investigated a population‐based cohort to determine white matter diffusion properties 8 years after PFS.

**Methods:**

We used diffusion tensor imaging (DTI) and applied Tract‐Based Spatial Statistics for voxel‐wise comparison of white matter microstructure between 26 children with PFS and 27 age‐matched healthy controls. Age, gender, handedness, and hippocampal volumes were entered as covariates for voxel‐wise analysis.

**Results:**

Mean duration between the episode of PFS and follow‐up was 8.2 years (range 6.7–9.6). All children were neurologically normal, and had normal conventional neuroimaging. On voxel‐wise analysis, compared to controls, the PFS group had (1) increased fractional anisotropy in early maturing central white matter tracts, (2) increased mean and axial diffusivity in several peripheral white matter tracts and late‐maturing central white matter tracts, and (3) increased radial diffusivity in peripheral white matter tracts. None of the tracts had reduced fractional anisotropy or diffusivity indices in the PFS group.

**Significance:**

In this homogeneous, population‐based sample, we found increased fractional anisotropy in early maturing central white matter tracts and increased mean and axial diffusivity with/without increased radial diffusivity in several late‐maturing peripheral white matter tracts 8 years post‐PFS. We propose disruption in white matter maturation secondary to seizure‐induced axonal injury, with subsequent neuroplasticity and microstructural reorganization as a plausible explanation.

**Key Points**

- Children with PFS demonstrate extensive white matter changes on diffusion tensor imaging 8 years after the episode
- The topographic pattern of diffusion abnormalities suggests a developmentally based preferential susceptibility of late‐maturing white matter tracts to PFS‐induced damage
- A plausible explanation could be disruption in white matter maturation secondary to seizure‐induced axonal injury, with subsequent neuroplasticity and microstructural reorganization

Prolonged febrile seizures (PFS) are the most common cause of convulsive status epilepticus (CSE) in children.^1^ Febrile seizures have received considerable research interest owing to the long‐recognized, but yet unresolved, association between PFS and mesial temporal sclerosis/temporal lobe epilepsy.^2^, ^3^, ^4^, ^5^ Although the hippocampus has been the primary focus in the majority of studies investigating the consequences of prolonged seizures, acute seizure‐related injury has also been reported in extra‐hippocampal cortical and subcortical gray matter (GM), and white matter (WM).^6^, ^7^, ^8^ There are considerable data on the longer‐term evolution of hippocampal changes following PFS, but the longer‐term outcomes of WM changes have not yet been investigated.^3^, ^4^, ^5^

Diffusion tensor imaging (DTI), a quantitative magnetic resonance imaging (MRI) technique, can identify microstructural alterations that are not evident on conventional structural sequences. DTI measures water diffusion within brain tissue, which reflects the organizational integrity of WM architecture and characteristics of fiber bundles in vivo.^9^ It provides quantitative measures including fractional anisotropy (FA) as a normalized scalar measure of the degree of diffusion anisotropy (directionality), axial diffusivity (AD) as diffusion parallel to WM tracts, radial diffusivity (RD) as diffusion perpendicular to WM tracts, and mean diffusivity (MD) as the magnitude of overall diffusion. These measures have been shown to be useful markers of changes in axonal density and size, myelination, and fiber organization.^10^

Abnormal DTI findings such as reduced FA and increased mean diffusivity have been reported in several WM tracts in the absence of a structural lesion on MRI in adults and children with epilepsy.^11^, ^12^ Thus DTI analysis could provide insight into WM microstructure in children with PFS and normal structural MRI.

In a recent study, we performed longitudinal DTI analysis in the first year following PFS, and found widespread changes suggestive of reduced WM integrity at 1 and 6 months, with apparent normalization 12 months post‐PFS.^13^ The WM changes were seen in the absence of any hippocampal or extra‐hippocampal structural abnormalities. Whether these WM changes persist or evolve after 1 year post‐PFS is unknown, and therefore medium‐ to long‐term follow‐up is necessary to investigate the outcomes.

We used tract‐based spatial statistics (TBSS) in the current follow‐up study of a unique population‐based childhood CSE cohort to investigate WM diffusion properties 8 years post‐PFS compared to healthy controls.^1^

## Materials and Methods

### Participants

The data for the current study were collected as part of a larger follow‐up study investigating outcomes within 10 years after childhood CSE. All 56 children who were classified as having had PFS in the original study were considered for entry into the current study.^1^ PFS was defined as a single seizure, or two or more seizures between which consciousness was not regained, lasting at least 30 min, during a febrile illness (temperature above 38°C) in a previously neurologically normal child aged between 6 months and 5 years, and in the absence of a defined central nervous system infection. Age‐matched healthy children with no known history of seizures or neurologic/developmental problems were recruited as controls.

All study participants had a detailed neurologic and neuropsychology (using Wechsler Abbreviated Scale of Intelligence [WASI]) assessment; and information on general health, psychomotor development, learning and memory, and handedness obtained through parental interviews.

### Ethics

The study was approved by the Institute of Child Health/Great Ormond Street Hospital Research Ethics Committee. Informed written consent was obtained from each participant and/or parent in the study.

### MRI acquisition

Conventional MRI and DTI sequences were acquired on an Avanto 1.5 Tesla scanner (Siemens, Erlangen, Germany) for all children. An imaging protocol optimized for the visualization of mesial temporal structures was carried out, including a T_1_‐weighted three dimensional (3D) Fast Low Angle SHot (3D‐FLASH) sequence (echo time 4.94 msec, repetition time 11 msec, field of view 256 × 224 mm, flip angle 15 degrees, and 1 × 1 × 1 mm image resolution). Echo‐planar diffusion‐weighted images were acquired for an isotropic set of 20 noncollinear directions, using a weighting factor of b = 1,000 s/mm^2^, along with a T_2_‐weighted (b = 0) volume. This protocol was repeated three times in a single scan session, and the data were merged without averaging. Forty‐five contiguous axial slices of width 2.5 mm were imaged, using a field of view of 240 × 240 mm and 96 × 96 voxel acquisition matrix, for a final image resolution of 2.5 × 2.5 × 2.5 mm. Echo time was 89 msec and repetition time was 6,300 msec. We took all necessary precautions during image acquisition to ensure the quality of diffusion data. The data were acquired by a specialist radiographer with several years of experience in pediatric MRI in a specialist pediatric hospital setting. The images of each participant were checked carefully during acquisition, and the sequences were repeated if motion distortion was observed in any of them on visual inspection. Seven children in the PFS group and five controls required reacquisition diffusion sequences due to motion artifact.

### MRI processing

DICOM format image files obtained from the scanner were converted to NIfTI‐1 format using the TractoR software package.^14^ Diffusion data were pre‐processed to correct for eddy current‐induced distortions using tools in the FSL package v5.0.2.1 (http://www.fmrib.ox.ac.uk/fsl). The brain was segmented using FSL's brain extraction tool, and diffusion tensors were calculated using least squares fitting. Individual measures of anisotropy (FA) and diffusivity (MD, AD, and RD) were calculated and used for voxel‐wise analysis.

### Qualitative MRI assessment

All MRI scans were reviewed by two experienced pediatric neuroradiologists, with particular attention to mesial temporal sclerosis (MTS). Radiologic criteria used for the diagnosis of MTS were presence of reduced hippocampal volume, increased hippocampal T_2_ signal, and disturbed internal architecture.^15^

### Hippocampal volume measurements

The 3D‐FLASH images reformatted perpendicular to the long axis of the hippocampus were used for hippocampal volume measurement. Two trained observers (SP and MM) independently performed manual hippocampal region of interest (ROI) tracing, blind to all clinical details. Binary ROI masks were drawn separately for each hippocampus using a protocol based on previously published methods.^16^ The intraclass correlation coefficient (ICC) values for hippocampal segmentation were 0.94 for left and 0.95 for right, and 0.93 for left and 0.95 for right for inter‐rater and intra‐rater reliability, respectively. Intracranial volume (ICV) was calculated using brain extraction tool in FSL. For each subject, the hippocampal volumes calculated from ROIs of the two observers were averaged and the ICV‐corrected hippocampal volumes were used for further analysis.

### Tract‐based spatial statistics (TBSS)

Statistical analysis of the FA data was carried out using TBSS, part of FSL. TBSS is a robust technique for automated voxel‐wise analysis of multisubject data to compare their WM diffusion properties.^17^ It aligns all subjects’ FA maps into a common space, creates a mean FA skeleton representing the centers of all tracts common to the group, and then projects all subjects’ FA data onto the mean FA skeleton, which is fed into voxel‐wise analysis. The mean FA skeleton was thresholded at an FA value of 0.2 to eliminate skeletal remnants at the outer WM tissue where higher intersubject variability is likely to cause poor alignment. We opted to generate a study‐specific target image for nonlinear registration, as adult‐derived standard‐space FA image used in TBSS may be inappropriate for pediatric data. Each subject's scan was visually inspected in FSLView, part of the FSL package, to ensure adequate registration. Other diffusivity measures (MD, AD, and RD) were also analyzed in TBSS using the “tbss_non_FA” script.

Voxel‐wise analysis was performed using the “randomise” tool in TBSS with 5,000 permutations and diffusion measures compared between the PFS group and controls. As age, gender, and handedness can influence diffusion parameters, they were entered as covariates for voxel‐wise analysis. In addition, although none of the subjects had radiologic evidence of MTS, hippocampal volume was also entered as a covariate for voxel‐wise analysis to address the possibility that unrecognized subtle hippocampal damage could potentially influence WM diffusion properties. Threshold‐free cluster enhancement (TFCE) was performed to enhance cluster‐like structures without prior definition of a cluster‐forming threshold or extensive data smoothing. To control for multiple voxel‐wise comparisons, family‐wise error (FWE) correction was performed and the resulting significance threshold was p < 0.05. Results were visualized in FSLView as color‐coded masks superimposed on the MNI152 brain template and the mean FA skeleton. The most probable anatomic localization of each significant cluster was determined using Johns Hopkins University WM atlas tools in FSL.^18^ To determine the magnitude of changes in FA and diffusivity parameters between the PFS group and controls, information about significant clusters on TBSS was extracted using the “cluster” tool in FSL.

All statistical tests, other than TBSS, were performed using SPSS (IBM SPSS Statistics for Macintosh, v21.0.; IBM Corp, Armonk, NY, U.S.A.).

## Results

**Figure 1:**
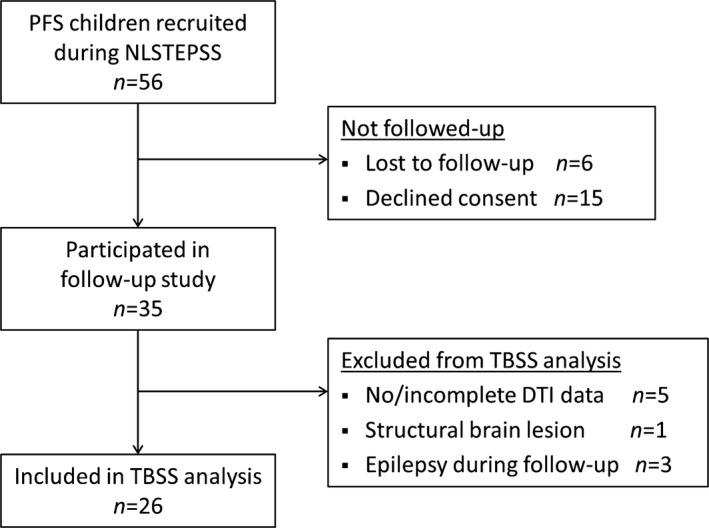
Flow diagram showing recruitment for the current study and reasons for noninclusion in the TBSS analysis.

Of the 56 children with an initial diagnosis of PFS, 35 (62.5%) consented to follow‐up. The reasons for loss to follow‐up and exclusion from current analysis are presented in Figure 1. Because our aim was to investigate WM microstructure in a homogeneous PFS cohort, we excluded three children who developed epilepsy during follow‐up and one child with MRI features of neurofibromatosis type 1. In addition, five children had to be excluded from the analysis because of incomplete DTI data. This includes one child who had evidence of unilateral MTS on conventional imaging, but for whom we could not acquire DTI data due to lack of cooperation. There was no significant difference in the median age at PFS (13 months vs. 16 months, Mann‐Whitney *U* p = 0.16) and seizure duration (60 min vs. 60 min, Mann–Whitney *U* p = 0.73) of children included in TBSS analysis and of those excluded. The mean interval between the episode of PFS and follow‐up was 8.2 years (range 6.7–9.6).

### Subject demographics

Twenty‐six children with PFS (11 male, mean age 9.9 years [standard deviation (SD) 1.72], 20 right‐handed) and 27 age‐matched controls (12 male, mean age 10.1 years [SD 1.74], 22 right‐handed) were included in the analysis. Conventional structural neuroimaging was normal in all, and none had neurologic or cognitive problems. None of the children in our PFS cohort had a clinical/genetic diagnosis of Dravet syndrome.

### Hippocampal structure and volume

None of 53 subjects included in the analysis had evidence of MTS. There was no significant difference in mean ICV‐corrected hippocampal volume between the PFS group and controls on the left (mean difference 38 mm^3^, p = 0.63 on *t*‐test) or the right side (mean difference 55 mm^3^, p = 0.53 on *t*‐test).

### TBSS analysis

No significant correlations were observed between the mean values for FA, MD, AD, and RD over the entire WM skeleton and the hippocampal volumes, in either the PFS group or controls (p > 0.05 for all). Likewise, we did not find significant correlation between the length of follow‐up and the mean values for FA, MD, AD, and RD.

**Figure 2:**
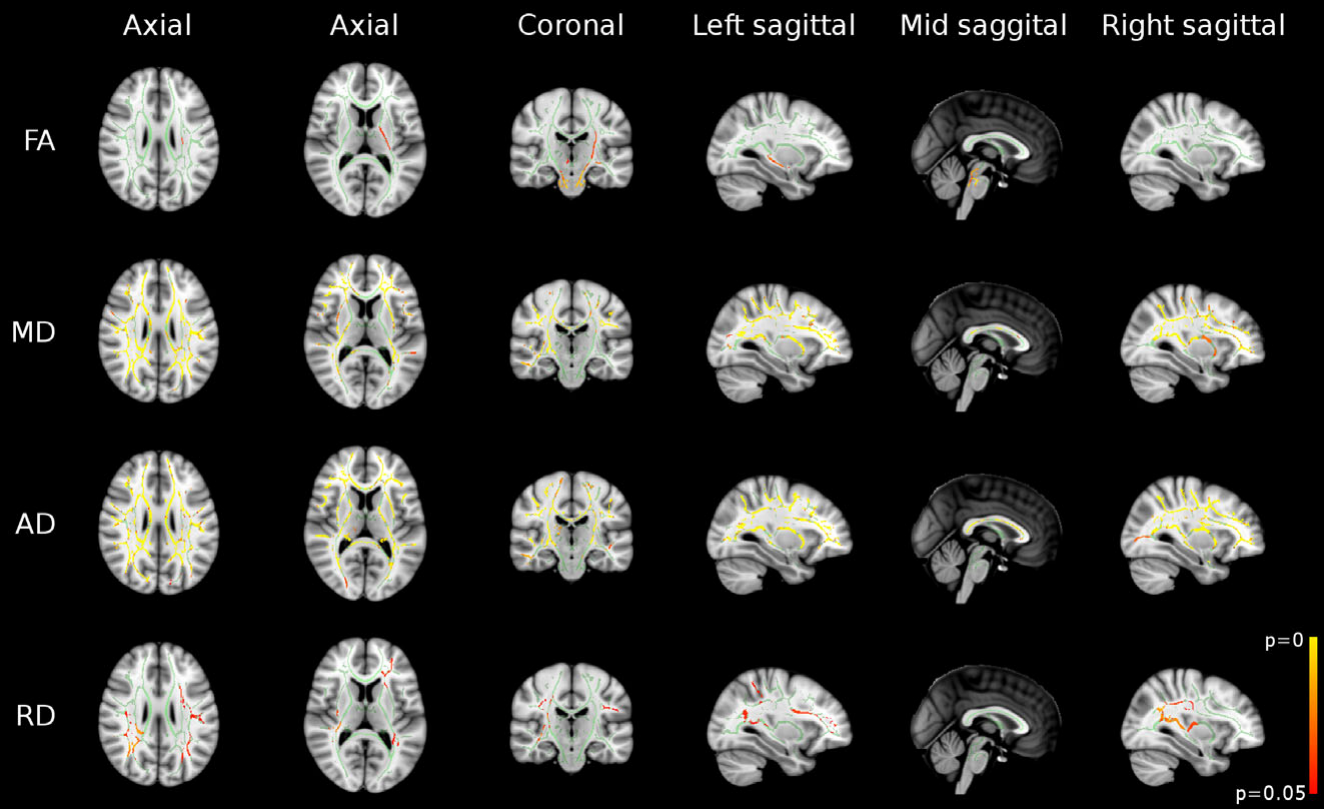
Results of whole‐brain TBSS analysis revealed widespread differences between the PFS group and controls in FA, mean diffusivity (MD), axial diffusivity (AD), and radial diffusivity (RD) maps. Regions in green represent the mean FA skeleton, and therefore no significant difference between the two groups. Regions in red‐yellow represent significant increases in the PFS group (p < 0.05, FWE corrected).

On voxel‐wise analysis, widespread differences in anisotropy and diffusivity measures were observed between the PFS group and controls (Fig. 2). Compared to controls, the PFS group had increased FA in caudal central WM tracts, and increased MD, AD, and RD in peripheral WM tracts.

In the PFS group, bilateral increases in FA were observed in the cerebral peduncles, corticospinal tracts, superior cerebellar peduncles, middle cerebellar peduncles, and the pontine crossing tract of middle cerebellar peduncle (Fig. 2, first row; Table 1). FA was also increased in the left superior corona radiata, posterior limb of internal capsule, and the crus fornix.

**Table 1:** White matter tracts showing significantly increased fractional anisotropy (FA) in the PFS group compared to the control group

WM tract		PFS group mean (SD)	Controls mean (SD)	Percent difference	Cluster size (voxels)	Coordinates (*x*,* y*,* z*)
Superior corona radiata	L	0.594 (0.062)	0.574 (0.062)	3.5	100	−27, −20, 21
Posterior limb of internal capsule	L	0.636 (0.061)	0.616 (0.062)	3.3	402	−16, −8, 9
Crus fornix	L	0.519 (0.074)	0.484 (0.069)	7.3	121	−28, −27, ‐3
Cerebral peduncle	L	0.614 (0.115)	0.584 (0.111)	5.1	234	−10, −18, −18
	R	0.611 (0.103)	0.588 (0.101)	3.8	109	11, −19, −19
Corticospinal tract	L	0.516 (0.073)	0.484 (0.073)	6.6	206	−7, −27, −27
	R	0.487 (0.071)	0.450 (0.072)	8.2	180	8, −26, −26
Superior cerebellar peduncle	L	0.610 (0.091)	0.580 (0.089)	5.0	53	−4, −30, −17
	R	0.562 (0.117)	0.531 (0.122)	5.1	68	7, −36, −25
Middle cerebellar peduncle		0.487 (0.110)	0.439 (0.101)	10.8	202	−10, −22, −33
					267	11, −33, −28
Pontine crossing tract		0.477 (0.055)	0.422 (0.058)	12.9	236	4, −30, −28

Cluster size = number of voxels in the largest cluster showing significant difference (p < 0.05, corrected for multiple comparisons); *x*,* y*,* z* = coordinates in Montreal Neurological Institute (MNI) space (mm).

There were widespread increases in MD and AD in the PFS group compared to controls (Fig. 2, second & third row; Tables 2 and 3). These were predominantly bilateral involving the corpus callosum, superior and inferior longitudinal fasciculi, inferior frontooccipital fasciculi, corona radiata, posterior thalamic radiations, retrolenticular part of internal capsules, external capsules, and cingulate gyri. MD was also increased in right anterior and posterior limbs of the internal capsule. In addition, AD increase was also seen in the posterior limb of internal capsules, cerebral peduncles, and crus fornices bilaterally, and in the right anterior limb of internal capsule.

**Table 2:** White matter tracts showing significantly increased mean diffusivity (MD) in the PFS group compared to the control group

WM tract		PFS group mean (SD) (×10^−4^ mm^2^/s)	Controls mean (SD) (×10^−4^ mm^2^/s)	Percent difference	Cluster size (voxels)	Coordinates (*x*,* y*,* z*)
Anterior corona radiata	L	7.77 (0.47)	7.53 (0.46)	3.2	1,067	−22, 18, 12
	R	7.73 (0.45)	7.54 (0.45)	2.5	1,038	19, 18, 33
Superior corona radiata	L	7.62 (0.39)	7.41 (0.39)	2.8	889	−26, 12, 24
	R	7.60 (0.39)	7.41 (0.37)	2.6	909	23, −24, 41
Posterior corona radiata	L	8.23 (0.60)	7.97 (0.52)	3.3	609	−22, −50, 30
	R	8.27 (0.60)	7.90 (0.50)	4.7	781	27, −26, 23
Superior longitudinal fasciculus	L	7.70 (0.51)	7.45 (0.48)	3.4	1,187	−37, −13, 26
	R	7.71 (0.51)	7.44 (0.46)	3.6	1,201	34, −28, 34
Sagittal stratum	R	8.48 (0.57)	8.24 (0.55)	2.9	231	40, −41, −4
Splenium of corpus callosum		8.18 (0.74)	7.80 (0.64)	4.9	1,424	16, −41, 24
Body of corpus callosum		8.65 (1.13)	8.31 (1.00)	4.1	2,107	−11, 1, 28
Genu of corpus callosum		8.04 (0.65)	7.79 (0.55)	3.2	185	−14, 21, 21
					289	15, 25, 18
Anterior limb of internal capsule	R	7.11 (0.32)	7.00 (0.33)	1.6	70	23, 11, 17
Posterior limb of internal capsule	R	7.44 (0.32)	7.31 (0.29)	1.8	478	28, −24, 17
Retrolenticular part of internal capsule	L	8.18 (0.47)	8.01 (0.45)	2.1	272	−33, −38, 10
	R	8.24 (0.55)	7.98 (0.53)	3.3	640	36, −35, 7
Posterior thalamic radiation	L	8.56 (0.61)	8.26 (0.52)	3.6	447	−29, −65, 16
	R	8.45 (0.66)	8.10 (0.56)	4.3	585	40, −42, −3
External capsule	L	7.84 (0.43)	7.66 (0.41)	2.3	678	−30, −1, 11
	R	8.08 (0.53)	7.89 (0.45)	2.4	624	34, −13, 8

Cluster size = number of voxels in the largest cluster showing significant difference (p < 0.05, corrected for multiple comparisons); *x*,* y*,* z* = coordinates in MNI space (mm).

**Table 3:** White matter tracts showing significantly increased axial diffusivity (AD) in the PFS group compared to the control group

WM tract		PFS group mean (SD) (×10^−4^ mm^2^/s)	Controls mean (SD) (×10^−4^ mm^2^/s)	Percent difference	Cluster size (voxels)	Coordinates (*x*,* y*,* z*)
Anterior corona radiata	L	11.43 (1.06)	11.07 (0.99)	3.3	929	−15, 34, 12
	R	11.60 (1.13)	11.21 (1.12)	3.5	1,094	21, 22, 24
Superior corona radiata	L	11.69 (1.10)	11.31 (1.07)	3.4	776	−18, −9, 42
	R	11.76 (1.15)	11.39 (1.12)	3.2	915	19, −1, 39
Posterior corona radiata	L	12.59 (1.10)	12.13 (1.09)	3.8	534	−29, −48, 25
	R	12.67 (1.22)	12.14 (1.18)	4.4	673	20, −46, 30
Superior longitudinal fasciculus	L	11.21 (1.12)	10.77 (1.07)	4.1	1,002	−32, −10, 28
	R	11.25 (1.15)	10.77 (1.06)	4.5	1,088	36, −3, 27
Sagittal stratum	L	12.63 (1.19)	12.34 (1.00)	2.4	95	−36, −16, −10
	R	13.19 (1.28)	12.80 (1.20)	3.0	200	41, −42, −4
Cingulum	L	12.16 (1.39)	11.55 (1.25)	5.3	75	−10, −26, 32
	R	10.84 (1.11)	10.37 (1.11)	4.5	55	9, −29, 36
Splenium of corpus callosum		15.77 (2.49)	15.08 (2.45)	4.6	1,669	11, −39, 24
Body of corpus callosum		15.94 (2.80)	15.32 (2.70)	4.0	2,851	7, −5, 25
Genu of corpus callosum		15.20 (2.30)	14.64 (2.23)	3.8	1,368	9, 28, 9
Anterior limb of internal capsule	L	12.40 (1.38)	12.11 (1.27)	2.4	103	−16, −3, 11
	R	12.76 (1.17)	12.43 (1.13)	2.7	385	18, 8, 10
Posterior limb of internal capsule	L	13.47 (0.94)	13.24 (0.92)	1.7	540	−22, −12, 15
	R	13.62 (1.09)	13.38 (1.07)	1.8	638	21, −9, 13
Retrolenticular part of internal capsule	L	13.59 (1.21)	13.26 (1.16)	2.5	300	−29, −34, 17
	R	13.48 (1.25)	13.09 (1.20)	3.0	536	27, −23, 8
Posterior thalamic radiation	L	14.37 (1.44)	13.81 (1.42)	4.1	661	−30, −64, 15
	R	14.02 (1.54)	13.43 (1.52)	4.4	566	33, −40, 14
External capsule	L	11.29 (1.09)	10.95 (1.02)	3.1	929	−31, −7, 13
	R	11.25 (1.17)	10.94 (1.10)	2.8	802	32, −2, 12
Crus fornix	L	13.06 (1.67)	12.57 (1.54)	3.9	102	−27, −33, 3
	R	13.38 (2.48)	12.79 (1.63)	4.6	84	29, −26, −4
Cerebral peduncle	L	15.80 (1.70)	15.45 (1.57)	2.3	125	−10, −16, −18
	R	14.56 (1.40)	14.31 (1.37)	1.7	87	15, −11, −8

Cluster size = number of voxels in the largest cluster showing significant difference (p < 0.05, corrected for multiple comparisons); *x*,* y*,* z* = coordinates in MNI space (mm).

Unlike MD and AD, regions of RD increase in the PFS group were less extensive, and limited to peripheral WM tracts such as the superior and inferior longitudinal fasciculi, inferior frontooccipital fasciculi, superior and posterior corona radiata, and posterior thalamic radiations (Fig. 2, last row; Table 4).

**Table 4:** White matter tracts showing significantly increased radial diffusivity (RD) in the PFS group compared to the control group

WM tract		PFS group mean (SD) (×10^−4^ mm^2^/s)	Controls mean (SD) (×10^−4^ mm^2^/s)	Percent difference	Cluster size (voxels)	Coordinates (*x*,* y*,* z*)
Anterior corona radiata	L	6.13 (0.62)	5.84 (0.62)	5.0	321	−21, 29, 11
Superior corona radiata	L	5.93 (0.55)	5.70 (0.56)	4.0	338	−27, −8, 32
	R	5.29 (0.64)	5.08 (0.64)	4.1	118	23, −24, 34
Posterior corona radiata	L	6.29 (0.74)	5.93 (0.65)	6.1	66	−29, −57, 19
	R	6.17 (0.77)	5.81 (0.69)	6.2	618	22, −46, 34
Superior longitudinal fasciculus	L	5.96 (0.61)	5.59 (0.58)	6.6	148	−39, −16, 26
	R	5.82 (0.59)	5.54 (0.57)	5.1	419	27, −32, 37
Sagittal stratum	R	6.34 (0.71)	6.04 (0.68)	5.0	66	37, −17, −9
Retrolenticular part of internal capsule	R	5.59 (0.65)	5.29 (0.61)	5.7	386	36, −35, 7
Posterior thalamic radiation	L	5.94 (0.72)	5.57 (0.67)	6.6	109	−31, −57, 11
	R	5.75 (0.79)	5.36 (0.68)	7.3	307	35, −44, 1
External capsule	R	6.51 (0.57)	6.28 (0.54)	3.7	232	35, −14, −5

Cluster size = number of voxels in the largest cluster showing significant difference (p < 0.05, corrected for multiple comparisons); *x*,* y*,* z* = coordinates in MNI space (mm).

None of the WM tracts had reduced FA or any of diffusivity indices in the PFS group.

## Discussion

To our knowledge, this is the first study to report on WM microstructure at medium‐ to long‐term follow‐up in a population‐based PFS cohort. The main findings from our study are that, compared to healthy controls, children who had PFS demonstrate the following: (1) FA increase primarily in caudal central WM tracts, (2) MD and AD increase in several peripheral WM tracts and rostral central WM tracts, and (3) RD increase limited to peripheral WM tracts, after a mean follow‐up of 8.2 years. The WM diffusion abnormalities were observed in the absence of hippocampal structural abnormalities.

Brain WM maturation in healthy children and adolescents follows predictable topographic and chronologic sequences, with myelination progressing from inferior to superior, from posterior to anterior, and from central to peripheral locations within the brain.^19^, ^20^, ^21^, ^22^ Therefore, myelination occurs in brainstem WM before cerebral WM, in central WM before peripheral WM, and in projection and commissural pathways before association pathways. The rate of maturation of WM tracts, however, is not linear, with a steep increase in the first year after birth and a slower rate in the following years.^19^, ^20^, ^23^, ^24^, ^25^ In addition, WM tracts less mature at birth (e.g. peripheral WM) have a faster rate of maturation in early childhood compared to those more mature at birth (e.g. corticospinal tracts), and therefore may be more vulnerable to insults during this period.^19^, ^23^, ^24^, ^26^, ^27^

**Figure 3:**
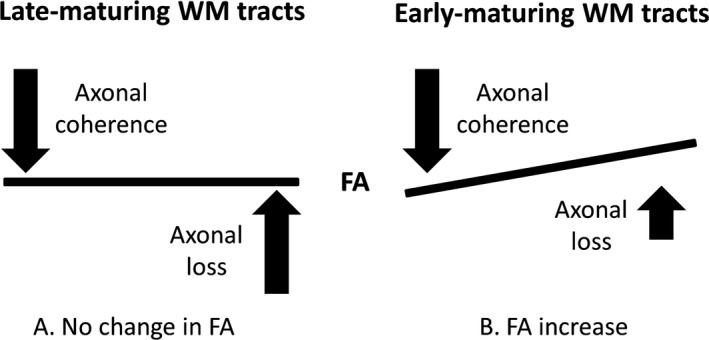
Illustration depicting the balance between axonal loss and axonal coherence, and their influence on FA. Although increased axonal coherence with modest axonal loss in early‐maturing WM tracts results in FA increase, the increased axonal coherence is counterbalanced by a greater axonal loss in late‐maturing WM tracts with no change in FA.

The striking observation in this study is the widespread increase in AD in the PFS group compared to controls. A possible mechanism for AD increase is greater coherence and alignment of the axons.^21^, ^25^, ^28^, ^29^ Alongside this, increases in MD suggest disruption of WM microstructure perhaps as a result of decreased axonal or myelin content. These two factors, axonal/myelin density and axonal alignment, contribute to FA in WM tracts. Figure 3 illustrates the balance between these two factors and shows how changes in FA can be explained in terms of them. For example, based on this model we suggest that an increase in axonal coherence (tending to increase FA) outweighs the more modest loss of axons (tending to reduce FA) leading to an overall increase in FA. However, in the late‐maturing tracts the increase in axonal coherence is counter‐balanced by a greater reduction in axonal density leading to no change in FA.

Of interest, the topographic pattern of diffusion abnormalities seen in the PFS group in our study relates to normal WM maturation in childhood. Early maturing WM tracts such as the corticospinal tracts, cerebral peduncles, and middle cerebellar peduncles, had increased FA in the PFS group, which we suggest is due to a combination of increased axonal alignment and modest axonal loss, compared to controls. In contrast, most late‐maturing WM tracts had evidence of disrupted maturation with increases in MD, AD, and RD, without a significant change in FA. In the PFS group, an increase in AD was observed in all association and commissural tracts, and in rostral projection fibers. Therefore, it could be hypothesized that an insult during a “vulnerable period” for late‐maturing WM tracts may be responsible for disrupted WM tract maturation in the PFS group. A recent report by Lee et al.^27^ of preferential susceptibility of late‐maturing WM tracts to damage in patients with mesial temporal lobe epilepsy also supports this hypothesis.

In our previous study of longitudinal WM diffusion changes following PFS, we found widespread reductions in FA in several WM tracts at 1 and 6 months post‐PFS, without a significant change in MD.^13^ The FA reductions were related predominantly to reductions in AD at 1 month, and increases in RD at 6 months post‐PFS. Although the reductions in AD almost disappeared, RD increases were more widespread at 6 months post‐PFS. No significant differences between the PFS group and controls in any of the diffusion parameters were observed at 1 year post‐PFS. Seizure‐related temporary disruption of WM development was proposed as the most biologically plausible explanation for the findings. Data from the current study in a similar cohort suggest that despite apparent normalization of WM DTI metrics at 1 year, alteration in WM microstructure compared to controls is evident about 8 years after PFS, and that this alteration is accompanied by apparent increases in the coherence of the remaining WM structure. Similar findings of FA increase, often accompanied by an increase in AD, have also been reported several months after brain insult in the form of brain surgery, stroke, and traumatic brain injury.^30^, ^31^, ^32^, ^33^ It is tempting to suggest that this reorganization of the remaining WM structure is the brain's attempt to maintain efficient organization in the face of an event disruptive to the normal trajectory of maturation, and one that has served to maintain neurologic and cognitive function as observed in our cohort.

The biophysical characteristics of WM affecting the diffusivity parameters on in vivo DTI are likely to be complex and multifarious.^34^, ^35^, ^36^, ^37^ Although FA (measure of intravoxel directional coherence of water diffusion) and MD (measure of magnitude of diffusion) are sensitive to changes in underlying tissue diffusion properties, changes in these metrics are not specific to particular elements of the underlying microstructure (i.e., number of axons, axon density, axon diameter, myelin density; all of which influence diffusion properties). The assumption that AD (i.e., axial/longitudinal/parallel diffusivity) is determined primarily by the intrinsic characteristics of axons and axonal integrity, and RD (i.e., radial/transverse/perpendicular diffusivity) by myelination, is overly simplistic, given that the relative contribution of WM tissue components to the diffusivity measures are yet to be accurately determined.^34^, ^35^ The interpretation of DTI metrics is even more ambiguous in regions of crossing fibers, where their values are likely to be confounded by changes in the relative fractions of the multiply oriented fiber populations.^36^, ^37^, ^38^ Although we cannot exclude the possibility that the observed DTI changes in the PFS group in our study could represent preexisting WM structural alterations in children predisposed to having PFS, the distribution, pattern, and the evolution of DTI changes in the context of previously reported short‐term changes suggest compensatory WM microstructural reorganization with increased axonal coherence as a more plausible explanation.

Another plausible explanation for the diffusion characteristics we observed long term following PFS is that they preexist PFS. We note from our short‐term follow‐up study that FA decreased at 1 month and 6 months post‐PFS and then normalized at 1 year with no evidence for FA increases as observed here.^13^ Similarly, AD was decreased at 1 and 6 months post‐PFS with no evidence for AD increases as observed in the present study. We therefore think that the diffusion changes we observed at long‐term follow‐up reported here with respect to healthy age‐matched controls are unlikely to preexist PFS, but it is impossible to completely rule this out in the absence of pre‐PFS imaging data, which is clearly difficult to obtain. Future studies using new multi‐shell diffusion MRI techniques that can quantify orientation dispersion unambiguously may shed further light on the mechanisms responsible for the diffusion changes observed in our study.^39^

The results of our study need to be interpreted with due consideration to some limitations. A longitudinal study design could have provided a better description of the evolution of WM diffusion changes following PFS. We, nonetheless, have referred to the results from our previous study on a comparable cohort, but with a shorter follow‐up, to complement our data and interpret the findings.^13^ A possible limitation of TBSS is misregistration of individual scans to the common template for group analysis, which may be an issue particularly for structurally abnormal conventional scans. However, all children in our study had structurally normal conventional scans, and we used a study‐specific target template instead of the adult‐derived standard template for nonlinear registration and also confirmed adequate registration by visual inspection. Finally, although our sample size is modest, we used an unselected, homogeneous PFS cohort and age‐matched controls to minimize the likelihood of potential bias and to allow for generalizability of our findings.

We report for the first time medium‐ to long‐term microstructural alterations in WM tracts following PFS in a population‐based cohort. We found increased FA in early maturing central WM tracts and increases in AD and MD with/without increased RD in several late‐maturing peripheral WM tracts 8 years after PFS. We propose temporary disruption in WM maturation secondary to PFS‐induced axonal injury, and subsequent compensatory microstructural reorganization with increased axonal coherence as a plausible explanation for the observed diffusion abnormalities. These findings appear to be consistent with the maintenance of neurologic and cognitive function observed in this cohort.

## Funding

This research was funded by grants from the BUPA Foundation (BUPA 22094612), The Academy of Medical Sciences and Wellcome Trust (SGCL1Chin), and the National Institute for Health Research Biomedical Research Centre at Great Ormond Street Hospital for Children NHS Foundation Trust and University College London. S.P. was supported by funding from Young Epilepsy.

## Disclosure

None of the authors has any conflict of interest to disclose. All authors confirm that they have read the Journal's position on issues involved in ethical publication and affirm that this report is consistent with those guidelines.
